# Injuries as a result of treatment of tibial fractures in children: Claims for compensation submitted to the Patient Insurance Center in Finland

**DOI:** 10.1080/17453670902805080

**Published:** 2009-02-01

**Authors:** Sauli Palmu, Reijo Paukku, Mervi K Mäyränpää, Jari Peltonen, Yrjänä Nietosvaara

**Affiliations:** ^1^Children’s Hospital, Helsinki University Central HospitalHelsinkiFinland; ^2^Patient Insurance CenterHelsinkiFinland

## Abstract

**Background and purpose** Tibial fractures comprise 10% of all fractures in children. To our knowledge there have been no previous reports of treatment injuries in these fractures. We analyzed compensation claims concerning treatment of these fractures in Finland. We used this information to determine preventable causes of treatment injuries.

**Material and methods** In Finland, the Patient Insurance Center (PIC) provides financial compensation for patients who have sustained an injury in connection with medical treatment or operation. We retrospectively analyzed all claims for compensation arising from treatment of tibial fractures in children that had been received by the PIC between 1997 and 2004. The mode of treatment, complications, and permanent sequelae were assessed. We also estimated the number of avoidable treatment injuries.

**Results and interpretation** The PIC received 50 claims for compensation during the 8-year study period. The claims were based on the following issues: pain, incorrect diagnosis and treatment, permanent disability, extra treatment expenses, inappropriate behavior of the medical personnel, and loss of income of the parents. 35/50 claims had received compensation, of which 32 were related to the treatment and 3 to infections. The treatment injuries that had led to compensation comprised a delay in diagnosis and treatment in 15 patients, inappropriate casting in 9, inappropriate operative treatment in 5, and other causes in 3 patients. An unsatisfactory standard of treatment and missed diagnosis were the most common reasons for compensation. In restrospect, all but 1 of the 35 injuries that had led to compensation were considered to be avoidable.

## Introduction

The Finnish Patient Insurance Center (PIC) provides financial compensation for patients, who have sustained an injury in connection with medical treatment or operation in accordance with the Finnish Patient Injuries Act, which came into force on May 1, 1987. PIC is the only institution of its kind in Finland and insurance companies that provide patient insurances are all members of PIC. Patients can apply for compensation without having to prove that the injury was the result of fault or neglect. Around 7,000 claims are filed annually, and approximately 30% of these lead to compensation. Treatment injuries are the most usual form of injury receiving compensation. Other injuries that can lead to compensation are infection (accidental or equipment-related) and unreasonable injuries (a disproportion exists in the severity of the injury being treated and the complication of the treatment). To our knowledge, there have been no previous reports on treatment injuries in children’s fractures.

Tibial fractures account for about 10% of all fractures in children under the age of 17 years (Worlock and Stower 1986, Landin 1997, Tiderius et al. 1999). Traditionally, tibial shaft fractures have been treated by cast immobilization (Blount 1955, Shannak 1988, O'Brien et al. 2004), although operative treatment has shown increasing popularity (Goodwin et al. 2005, Kubiak et al. 2005). We are unaware of any prospective randomized studies comparing operative and nonoperative treatment in tibial fractures in children. The treatment methods may vary between different institutions and countries (Grimard et al. 1996), but it is generally accepted that open fractures and displaced intraarticular proximal and distal fractures are an indication for operative treatment (O'Brien et al. 2004). Complications related to nonoperative treatment include compartment syndrome, skin ulcerations, peroneal palsy, delayed union, and malunion (Hansen et al. 1976). In addition to the complications of nonoperative treatment mentioned here, surgical treatment can lead to iatrogenic neurovascular injuries and infections (Kubiak et al. 2005).

We analyzed patient compensation claims concerning tibial fracture treatment of children in Finland. We have used this information to outline preventable causes of treatment injuries with a view to reducing their occurrence.

## Patients and methods

Information on children (< 17 years of age) in Finland, and in the city of Helsinki in particular, was obtained from national register data. The number of children who received inpatient treatment for tibial fractures (excluding tibial eminentia avulsions) in Finland between 1997 and 2004 was calculated from hospital discharge registers. The distribution of inpatient care in different level institutions was also registered. The total incidence of fractures in the census population of the city of Helsinki (including patients who were not admitted to the hospital) was assessed prospectively over 1 year, starting from February 2005.

The data concerning claims for compensation regarding tibial fractures in children during the study period were obtained from the registers of the PIC. In addition to demographic data, a description of the treatment injury was requested.

**Figure 1.**
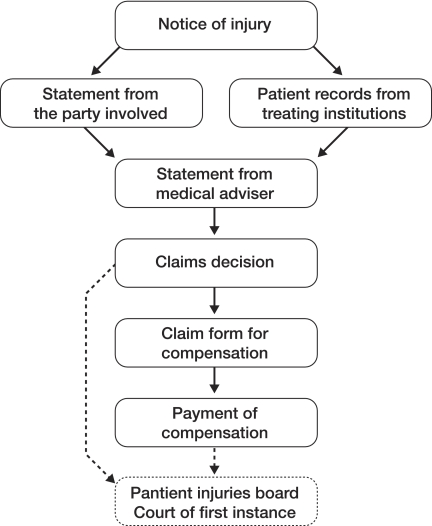
The claims-handling process of the Patient Insurance Center.

The compensation claims had been filed by the parents.Whether or not a compensatable treatment injury had occurred was evaluated by a medical adviser on the basis of the medical records available. The final decision about the compensation was made by the PIC (Figure 1).

In the present study, an independent observer (a consultant pediatric orthopedic surgeon, RP, who was not involved in the treatment of patients or in the handling of claims) retrospectively analyzed all patient claims and decisions concerning treatment made during the study period. Patient treatment files, statements, and decisions about compensation were re-evaluated. Age, sex, and type and location of the fracture were recorded. Trauma energy was graded either as high (e.g. traffic accident), moderate (e.g. sporting injuries), or low (e.g. falling on level ground). Mode of treatment, complications, and permanent sequelae were assessed. Reasons for the claim and for the compensations were also recorded. The number of compensations for the patients studied was obtained from PIC. The number of avoidable treatment injuries was estimated and a treatment protocol for tibial fracture treatment was constructed.

### Statistics

Statistical analysis was performed using SPSS 15.0 software. The 95% confidence interval (CI) for incidence was calculated using the Poisson distribution.

## Results

During the 8-year study period, the number of children in the country who were aged ≤ 17 varied between 1.04 million and 1.09 million. According to register data, the national incidence of tibial fractures (n = 5,908) in children who required hospital treatment was 0.69 per 1,000 (CI: 0.68–0.71) during the study period. Most of these children were treated in university hospitals (n = 2,337; 40%) or central hospitals (n = 2,280; 39%), and a minority in district hospitals (n = 898; 15%), healthcare center hospitals (n = 345; 6%), or private institutions (n = 48, 1%). The total incidence of tibial fractures in children in the census population, derived from the 12-month survey in the city of Helsinki, was 1.0/1,000.

During the 8-year period covered by the study, a compensation claim was filed for 36 patients with tibial fractures requiring inpatient care (0.6%). Claims were directed to treatment given in university hospitals (16), central hospitals (15), and district hospitals (5). 14 additional claims were filed concerning outpatient treatment (healthcare centers (12), central hospitals (1), and private institutions (1)).

The mean age of these 50 patients was 11 years; 12 (3–16) years for university hospitals, 13 (0–16) years for central hospitals, 10 (5–15) years for district hospitals, 7 (1–14) years for healthcare centers, and 3 years for private institutions. 32 children were boys. The right side was injured in 26 cases, the left side in 23, and 1 patient had a bilateral fracture. The fracture location was diaphyseal in 25 patients, distal metaphyseal in 12, proximal metaphyseal in 7, and intraarticular in 6. The physis was involved in 7 patients (14%). 4 fractures were open and 1 patient had multiple fractures. There was 1 vascular injury in connection with a proximal metaphyseal tibial fracture (the patient had been in a high-energy motocross accident). 5 patients suffered from compartment syndrome. There were no pathological fractures in this series. The trauma energy was considered high in 16 patients, moderate in 18, and low in 16.

**Figure 2.**
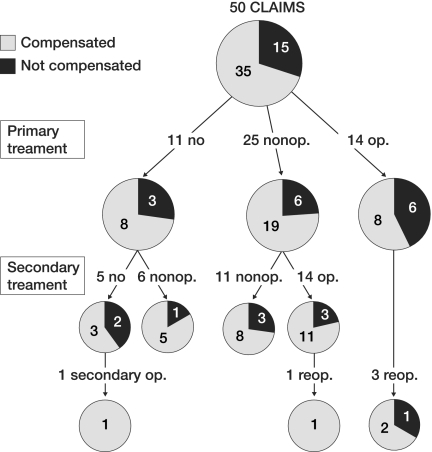
Compensation rates by method of treatment after pediatric tibial fracture treatment. no: no treatment initially; nonop.: nonoperative treatment; op.: operative treatment.

Primary treatment was performed by cast immobilization in 25 patients and by operative means in 14. The fracture diagnosis was initially missed in 11 patients: 1 patient was later operated, 6 were treated by casting, and 4 did not need any treatment at the time of correct diagnosis (Figure 2). Primary operative treatment included 5 screw fixations, 3 flexible intramedullary (IM) nailing, 3 rigid IM nailing, 1 plate-osteosynthesis, 1 external fixation, and 1 bio-absorbable pinning. 3 of these patients were reoperated: 1 flexible nailing was converted to rigid IM nailing, and 1 rigid IM nail and 1 plate osteosynthesis were both converted to external fixation. 14/25 of the primarily nonoperatively treated patients were eventually operated.

5 patients had more than one complication. Of 31 complications, 26 were regarded as being avoidable in the present re-evaluation (Table 1). Those that were classified as unavoidable were 3 infections related to operative treatment, 1 malunion, and 1 decubitus ulcer due to casting.

**Table 1.** Complications (n = 37) in 31/50 patients who claimed compensation after treatment. The numbers of cases who received compensation are given in parentheses

Complication	Treatment	
	operative	nonoperative
Infection	8 (6)	2 (2)
Skin ulceration		
casting		7 (7)
tourniquet	1 (1)	
Compartment syndrome	6 (3)	
Nerve palsy		
peroneal	5 (5)	
ulnar	1 (1)	
Popliteal artery injury	1 (1)	
Malunion		4 (3)
Nonunion		2 (1)
Total	22 (17)	15 (13)

Permanent sequelae of the treatment were seen in 12 patients: 5 malunions, 3 contractures of the ankle or the subtalar joint, 2 peroneal nerve palsies, 1 premature physeal closure, and 1 skin defect treated by plastic surgery. According to the graduated disability scale used in Finland (implemented in units of 5 from 0–100%), one of these patients had 30% disability, two had 10%, and one had 5% disability.

The claims for compensation were focused on the following issues: pain (n = 30), insufficient diagnosis and treatment (n = 23), permanent disability (n = 24), extra treatment expenses (n = 17), inappropriate behavior of the medical personnel (n = 1), and loss of income of the parents (n = 1). The number of issues claimed for per file was 1 in 19 cases, 2 in 19 cases, 3 in 13 cases, and 4 in 1 case.

35/50 claims were granted compensation, 32 of which were related to the treatment and 3 to infections (Table 2). There was no difference in distribution of fracture location regarding whether or not the claims were awarded compensation. Inadequate casting injuries included 6 decubitus ulcers, 5 of which were around the ankle. Compensated infection injuries occurred in 3 operatively treated cases: bioabsorbable pinning of a tibial tuberosity fracture, rigid IM nailing of a shaft fracture, and screw fixation of a triplane fracture. The infection injury in the triplane ankle fracture patient also received compensation as an unreasonable injury: 4 soft tissue revisions were needed after the primary fracture treatment. In 3 patients suffering from compartment syndrome, there was an unnecessary delay in fasciotomy. In 15 cases, the PIC did not award compensation for the injuries (Table 3).

**Table 2.** Details of the causes of the 32 treatment injuries that led to compensation

	n	avoidable
Delay	15	14
diagnosis	11	10/11
fasciotomy	3	yes
remanipulation	1	yes
Casting technique	9	8
decubitus heel	4	yes
decubitus (ankle)	1	yes
decubitus (combined)	1	yes
skin laceration (cast removal)	1	yes
malunion	2	1/2
Operative technique	6	6
iatrogenic peroneal palsy	2	yes
inadequate implant	2	yes
inadequate reduction	1	yes
skin necrosis caused by tourniquet	1	yes
Patient position during anesthesia	2	2
ulnar palsy	1	yes
peroneal palsy	1	yes

**Table 3.** Characteristics of 50 claims for compensation concerning injuries resulting from treatment of tibial fractures in children, listed according to type of institution, and their rates of compensation

	No. of claims	No. compensated
Private clinic	1	1
skin laceration during cast removal	1	1
Healthcare center	12	9
missed diagnosis (no radiographs)	7	5 **^a^**
fracture missed on radiographs	3	3
malunion	1	1
fracture could not be seen on radiograph	1	0
District hospital	5	4
decubitus (inadequate casting)	2	2
malunion	2	1 **^b^**
operative technique	1	1
Central hospital	16	12
decubitus (inadequate casting)	3	3
operative technique	2	2
infection	2	2
iatrogenic nerve injury	2	2
missed diagnosis (no radiographs)	1	1
delay in fasciotomy	1	1
malunion – no treatment	1	1
inadequate clinical examination	1	0 **^c^**
reduced range of motion	3	0
University hospital	16	9
infection	3	1
iatrogenic nerve injury	3	2
delay in fasciotomy	2	2
delay in arterioraphy	1	1
missed diagnosis (no radiographs)	1	1
decubitus (inadequate casting)	1	1
skin necrosis caused by tourniquet	1	1
remanipulation under anesthesia	1	0 **^d^**
compartment syndrome	1	0 **^e^**
pseudarthrosis	1	0 **^e^**
fracture missed on radiographs	1	0 **^d^**

**^a^** Injury regarded as tolerable.

**^b^** Malunion within acceptable limits.

**^c^** Minor injury.

**^d^** Treatment injury had no affect on the final outcome.

**^e^** Complication was caused by the primary injury.

The overall sum for compensations paid to the patients by PIC was approximately 137,000 euros. The average amount of compensation was 3,900 (200–43,867) euros per patient. PIC paid compensation for the following reasons: permanent disability (51,633 euros), pain (37,849 euros), cosmetic harm (26,155 euros), and extra treatment expenses. It is estimated by PIC that still another 30,000 euros will be paid out as compensation to the patients.

All but 1 of the claims that were awarded compensation (the infection injury of the triplane ankle fracture patient) were classified as being avoidable. The level of treatment in all but 4 of the 35 claims that were awarded compensation (3 infections related to operative treatment and 1 decubitus ulcer due to casting) was regarded as being below the standard expected of an experienced consultant. In 7 of the cases, the national treatment recommendations were not followed. After re-evaluating the patient treatment files, the statements from the experts, and the decisions about compensation, the independent observer ended up agreeing with the original decisions in all cases.

The use of radiographs had been inadequate in 11 cases: in 8 cases the diagnosis was missed because no radiographs were taken, and in three cases there was an error in interpreting the radiographs. In all but 1 of these cases, the primary treatment was given in healthcare centers.

The compensation rate was 9/16 in university hospitals, 12/16 in central hospitals, and 4/5 in district hospitals (Table 3).

## Discussion

To our knowledge, the frequency of treatment injuries relating to tibial fractures in children has not been reported before. According to our survey, the risk in Finland is low. The reasons for the claims for compensation varied in different treatment institutions. The most common reason for such claims after treatment in healthcare centers was a missed diagnosis. This can partly be explained by the fact that according to Finnish treatment guidelines, tibial fractures should be treated in surgical treatment units (Kunnamo et al 2006). This in turn means that general practitioners working in healthcare centers are not used to treating children with tibial fractures. The patients treated in healthcare centers were also younger, which may have complicated adequate fracture diagnosis (Irwin 2004). The reasons for claims for compensation after treatment given in hospitals were inadequate treatment rather than missed diagnosis. Although the Finnish Patient Insurance Act has made it obligatory for medical personnel to inform patients (or their parents) that they can apply for compensation if a treatment injury may have happened, all treatment injuries may not have ended up as claims to the PIC.

Complications of treatment occurred in two-thirds of the patients in this study. The complications reported were similar to those reported in earlier studies (Hansen et al. 1976, Shannak 1988, Kreder and Armstrong 1995, Karladani et al. 2000, Gordon et al. 2007). We were not able to evaluate the complication rate, since we are only aware of the complications that resulted in a claim for compensation. Casting injuries turned out to be an important subgroup among the injuries that led to compensation. Inappropriate casting resulted in 6 decubitus ulcers, 5 of them around the ankle. Decubitus ulcers should have been avoided with a better casting technique and better patient information.

In all but 1 of the claims that led to compensation, the injury was considered avoidable. Of these, 11 were cases with delayed diagnosis due to absence of radiographs or misinterpretation of radiographs. Wei et al. (2006) reported that 0.4% of tibial fractures were initially missed in radiographs, which was the case in 3 of our patients. In addition, 8 other patients in our study suffered from a delay in diagnosis because no radiographs were taken. It is noteworthy that all but one of these cases occurred in healthcare centers. According to Piene et al. (1991), there are about 60% more radiographs taken annually in Finland than in the other Nordic countries. On the basis of our study, we recommend the routine use of radiographs whenever tibial fracture is a possibility. This is especially important in healthcare centers that serve as primary screening points for further treatment.

Two-thirds of the claims in our series resulted in compensation. The average compensation rate for claims to the PIC is 1 out of 3 for all types of injuries. We conducted a second review of the claims for compensation submitted to the PIC and concluded that the original decisions about compensation were valid. The positive difference in the ratio of claims that led to compensation may mean that not all parents of children with tibial fracture filed a complaint about a treatment injury. There may have been additional treatment injuries that were not as severe as those analyzed in this study. According to the Finnish Patient Insurance Act, a compensatable treatment injury occurs if the treatment leading to a complication is below the standard of that expected for an experienced consultant. An unsatisfactory standard of treatment and missed diagnosis were the most common reasons for compensation in this study. In retrospect, all but 1 of the injuries that led to compensation could have been avoided, and the treatment was classified as being below the standard expected of an experienced consultant in all but 4 of the cases in which compensation was given.

According to the compensation data of the PIC, the average amount of compensation per treatment injury awarded by the PIC was approximately 3,900 euros. In addition, there were extra costs for the families and for society due to extra treatment.

Treatment injuries involving tibial fractures in children in Finland are rare, and most of them can be avoided. Our suggestions here will hopefully improve the treatment of tibial fractures in the future.
